# 

**Published:** 1900-01

**Authors:** 


### CONTENTS.

ORIGINAL COMMUNICATIONS.
Apollonia, the "Patron Saint of Dentistry." By C. N. PEIRCE, D.D.S., Philadelphia . . . 1
The Status of our Profession and its Educational Methods in England, as shown in Recent Publications. By WILLIAM H. TRUEMAN, D.D.S., Philadelphia . . . 3

REPORTS OF SOCIETY MEETINGS.
National Dental Association . . . 21
Academy of Stomatology . . . 32
The New York Institute of Stomatology . . . 39
Massachusetts Dental Society . . . 49

EDITORIAL.
Dental Epochs in the Nineteenth Century . . . 58

OBITUARY.
Dr. Benjamin H. Catching . . . 62
Academy of Stomatology—Resolutions upon the Death of Dr. Bonwill . . . 64

DOMESTIC CORRESPONDENCE.
Wisconsin State Board of Dental Examiners . . . 64

CURRENT NEWS.
A Bill for the Appointment of Dental Surgeons in the Army . . . 67
Thirteenth International Medical Congress . . . 68
Kentucky State Dental Association . . . 71
New York Odontological Society . . . 72
Second District Dental Society, State of New York . . . 72

